# Aquaculture and Florfenicol Resistance in *Salmonella enterica* Typhimurium DT104

**DOI:** 10.3201/eid1408.080329

**Published:** 2008-08

**Authors:** Peter Smith

**Affiliations:** * National University of Ireland, Galway, Ireland

**Keywords:** aquaculture, Salmonella typhimurium, drug resistance, microbial genetics, risk assessment, fish diseases, drug therapy, microbiology, letter

**To the Editor**: In June 2006, the World Health Organization (WHO), the Food and Agriculture Organization of the United Nations (FAO), and the World Organisation for Animal Health (OIE) convened an Expert Consultation to consider the risks to human health represented by the use of antimicrobial drugs in aquaculture. This would, therefore, appear to be an opportune time to reexamine some of the arguments that have been presented with respect to the assessment of these risks.

In their contributions to the debate regarding the risks associated with the use of antimicrobial agents in aquaculture, Angulo (*1*), Angulo and Griffin (*2*), Ribot et al. (*3*), and, more recently, Cabello (*4*) have argued that the available molecular evidence suggests that the *flo* gene that encodes chloramphenicol and florfenicol resistance in *Salmonella enterica* serovar Typhimurium DT104 (DT104) originally emerged in Japanese aquaculture and may have transferred horizontally from this host to DT104. This argument also appears in the report of the WHO/FAO/OIE consultation (ftp://ftp.fao.org/ag/agn/food/aquaculture_rep_13_16june2006.pdf). These authors (*1*–*4*) have based their argument on the assertions that florfenicol was first used in Japan and that *flo* gene–mediated resistance to this agent was first identified in bacteria isolated from Japanese fish farms.

In attempting to identify the date of the emergence of florfenicol resistance in Japanese aquaculture, Angulo and Griffin (*2*) state that florfenicol had been used in this country since the early 1980s. However, Schering Plough, the manufacturer of florfenicol, reports first marketing this agent for aquacultural use in Japan in 1990 (D. Schofield, pers. comm.), and this date for the introduction of florfenicol is also provided by Kim et al. (*5*). It should, however, be noted that the *flo* gene encodes resistance to both florfenicol and chloramphenicol and, therefore, *flo*-containing bacteria could be selected for by the use of either agent. As a consequence, arguments about the chronology of the first use of florfenicol may have limited relevance.

Florfenicol resistance in Japanese aquaculture (*5*) was first reported in strains of *Pasteurella piscicida* (now renamed *Vibrio damsela)*. The sequence of the gene that encoded florfenicol resistance in these strains (*6*) was demonstrated to have a 97% nucleotide sequence similarity to that found in strains of DT104 resistant to ampicillin, chloramphenicol, streptomycin, sulfonamide, and tetracycline (ACSSuT DT104) (*7*). The available data (*5*,*8*) allow a reasonably accurate estimate of the date when these florfenicol-resistant strains first emerged in Japanese aquaculture. The strains of florfenicol-resistant *P. piscicida* were first isolated in Japan in 1992 (*5*). However, a previous study (*8*) had demonstrated 100% susceptibility to florfenicol of *P. piscicida* strains isolated from 1989 through 1991. Because this study examined 175 *P. piscicida* strains isolated from fish farms distributed over a wide geographic area in Japan, the data it generated provide strong support for the conclusions that *flo-*mediated florfenicol resistance in *P. piscicida* first emerged in Japanese aquaculture in 1992 (*5*). However, the presence of the *floR* gene has been demonstrated in a strain of ACSSuT DT104 isolated in the United States in 1985 (*3*). Thus, the *floR* gene was present in DT104 strains isolated in the United States at least 7 years before the first bacteria containing this gene (*6*) were isolated from an aquaculture setting in Japan (*5*).

There are, however, data indicating that the *flo* gene was present in terrestrial bacteria associated with humans long before it was detected in multidrug -resistant DT104. A gene with a 95%–97% nucleotide identity with the *flo* gene of DT104 was detected in the Inc C plasmid R55 (*9*). This plasmid was originally identified in a chloramphenicol-resistant strain of *Klebsiella pneumonia*e isolated from a person in Paris in 1969 (*10*).

The earliest report of the isolation of a bacterium whose florfenicol resistance was encoded by a *flo* gene (*6*) and the earliest accession date for a *flo* gene sequence in GenBank (www.ncbi.nlm.nih.gov/Genbank) both related to *P. damsela* isolated from Japanese aquaculture. Publication and accession dates do not, however, constitute evidence of the date of the first isolation of a bacterium containing this gene. Analysis of the available chronological and molecular data presented here indicates that a variant of the *floR* gene in DT104 (*9*) was present in a terrestrial bacterium isolated in 1969 (*10*), 23 years before the first isolation, in 1992, of a bacterium associated with aquaculture that contained this gene (*5*). It further demonstrates that this gene was present in strains of DT104 isolated in 1985. Thus, these data provide no support for the arguments (*1*–*4*) that implicate aquacultural use of florfenicol or the subsequent occurrence of florfenicol-resistant *P. damsela* in the emergence of the *flo* gene in DT104.

## References

[1] Angulo F. Antimicrobial agents in aquaculture: potential impact on public health. Alliance for the Prudent Use of Antibiotics (APUA) Newsletter. 2000;18:1, 4.

[2] Angulo FJ, Griffin PM. Changes in antimicrobial resistance in *Salmonella enterica* serovar typhimurium. Emerg Infect Dis. 2000;6:436–8. 10905990 10.3201/eid0604.000429 PMC2640904

[3] Ribot EM, Wierzba RK, Angulo FJ, Barrett TJ. *Salmonella enterica* serotype Typhimurium DT104 isolated from humans, United States, 1985, 1990, and 1995. Emerg Infect Dis. 2002;8:387–91. 11971772 10.3201/eid0804.010202 PMC2730249

[4] Cabello FC. Heavy use of prophylactic antibiotics in aquaculture: a growing problem for human and animal health and for the environment. Environ Microbiol. 2006;8:1137–44. 10.1111/j.1462-2920.2006.01054.x 16817922

[5] Kim EH, Yoshida T, Aoki TBB. Detection of R plasmid encoded with resistance to florfenicol in *Pasteurella piscicida.* Fish Pathol. 1993;28:165–70.

[6] Kim E, Aoki T. Sequence analysis of the florfenicol resistance gene encoded in the transferable R-plasmid of a fish pathogen *Pasteurella piscicida.* Microbiol Immunol. 1996;40:665–9. 8908612 10.1111/j.1348-0421.1996.tb01125.x

[7] Bolton LF, Kelley LC, Lee MD, Fedorka-Cray PJ, Maurer JJ. Detection of multi-drug resistant *Salmonella enterica* serotype typhimurium DTI 04 based on a gene which confers cross-resistance to florfenicol and chloramphenicol. J Clin Microbiol. 1999;37:1348–51. 10203484 10.1128/jcm.37.5.1348-1351.1999 PMC84772

[8] Kim EH, Aoki T. Drug resistance and broad geographical distribution of identical R plasmids of *Pasteurella piscicida* isolated from cultured yellowtail in Japan. Microbiol Immunol. 1993;37:103–9. 8502175 10.1111/j.1348-0421.1993.tb03186.x

[9] Cloeckaert A, Baucheron S, Chaslus-Dancla E. Nonenzymatic chloramphenicol resistance mediated by IncC plasmid R55 is encoded by a *floR* gene variant. Antimicrob Agents Chemother. 2001;45:2381–2. 10.1128/AAC.45.8.2381-2382.2001 11451703 PMC90660

[10] Chabbert YA, Scavizzi MR, Witchitz JL, Gerbaud GR, Bouchaud DH. Incompatibility groups and the classification of fi-resistance factors. J Bacteriol. 1972;112:666–75. 4628744 10.1128/jb.112.2.666-675.1972 PMC251473

